# Hepatitis B Virus Exposure, Seroprotection Status, and Susceptibility in Health Care Workers From Lao People’s Democratic Republic: Cross-Sectional Study

**DOI:** 10.2196/65093

**Published:** 2024-12-17

**Authors:** Siriphone Virachith, Khanxayaphone Phakhounthong, Vilaysone Khounvisith, Mayfong Mayxay, Sengchanh Kounnavong, Somphou Sayasone, Judith M Hübschen, Antony P Black

**Affiliations:** 1 LaoLuxLab/Vaccine Preventable Diseases Laboratory, Institut Pasteur du Laos, Samsenthai Rd, P.O. Box 3560, Ban Kao-gnot, Vientiane, 01000, Lao People's Democratic Republic, 856 21 285 321; 2 Lao Tropical and Public Health Institute, Ministry of Health, Vientiane, Lao People's Democratic Republic; 3 Institute of Research and Education Development, University of Health Sciences, Ministry of Health, Vientiane, Lao People's Democratic Republic; 4 Lao-Oxford-Mahosot Hospital-Wellcome Trust Research Unit (LOMWRU), Microbiology Laboratory, Mahosot Hospital, Vientiane, Lao People's Democratic Republic; 5 Centre for Tropical Medicine and Global Health, Nuffield Department of Medicine, University of Oxford, Oxford, United Kingdom; 6 Saw Swee Hock School of Public Health, National University of Singapore, Singapore; 7 Department of Infection and Immunity, Luxembourg Institute of Health, Esch-sur-Alzette, Luxembourg; 8 School of Life Sciences, University of Westminster, London, United Kingdom

**Keywords:** hepatitis B, hepatitis D, health care workers, Laos, prevalence

## Abstract

**Background:**

Despite the high prevalence of chronic hepatitis B virus (HBV) infection in adults in Lao People’s Democratic Republic (Lao PDR), Lao health care workers (HCWs) have previously been shown to have low levels of protection against infection. Furthermore, the prevalence of hepatitis D virus (HDV), which increases disease severity in individuals infected with HBV, is not known in Lao PDR.

**Objective:**

This study aimed to estimate the exposure and seroprotection against HBV, as well as exposure to HDV, in Lao HCWs from 5 provinces.

**Methods:**

In 2020, a total of 666 HCWs aged 20 to 65 years from 5 provinces of Lao PDR were recruited, and their sera were tested by enzyme-linked immunosorbent assay to determine their HBV and HDV coinfection status.

**Results:**

HBV exposure, as indicated by the presence of anti–hepatitis B core antibodies, was 40.1% (267/666) overall and significantly higher for HCWs from Oudomxay province (21/31, 67.7%; adjusted odds ratio 3.69, 95% CI 1.68‐8.12; *P=*.001). The prevalence of hepatitis B surface antigen was 5.4% (36/666) overall and increased with age, from 3.6% (9/248) in those aged ≤30 years to 6.8% (8/118) in those aged ≥50 years. Only 28.7% (191/666) of participants had serological indication of immunization. We could find no evidence for HDV exposure in this study.

**Conclusions:**

The study found intermediate hepatitis B surface antigen prevalence among HCWs in Lao PDR, with no evidence of HDV coinfection. Notably, a significant proportion of HCWs remains susceptible to HBV, indicating a substantial gap in seroprotection against the disease.

## Introduction

Hepatitis B virus (HBV) is a major global public health concern that can cause chronic infection, cirrhosis, liver cancer, and death. HBV is transmitted by exposure to infected blood or other body fluids, such as semen and vaginal fluid during sexual intercourse, tattooing, unsafe medical practices, and mother-to-child routes during pregnancy or shortly after birth [1,2]. Most people infected with HBV are asymptomatic and may be unaware of being infected and the risk of transmitting the virus to others. Hepatitis D virus (HDV) requires HBV presence for its replication in hepatocytes. HBV-HDV coinfection greatly increases the risk of liver cirrhosis and hepatocellular carcinoma development [3,4].

For individuals with chronic infections, hepatitis B surface antigen (HBsAg; found on the surface of HBV and in serum) and anti–hepatitis B core (anti-HBc) antibody persist in their blood for life. Individuals who recover from a natural hepatitis B infection usually test positive for both anti–hepatitis B surface (anti-HBs) and anti-HBc antibodies. In contrast, those who respond to the hepatitis B vaccine will only show anti-HBs antibodies. Following vaccination with the hepatitis B vaccine, anti-HBs immunoglobulin G levels can be detectable for many years, typically lasting at least 5 to 10 years, and sometimes even longer [5].

The World Health Organization (WHO) estimated that in 2019, about 296 million people were living with chronic HBV infection with 820,000 deaths [6]. The highest prevalence of infection occurs in the WHO Western Pacific Region, with more than one-third of the HBV infections worldwide [6,7]. Within this region, Lao People’s Democratic Republic (Lao PDR) is considered to have intermediate to high endemicity of HBV infection, with HBsAg prevalence ranging from 5% to 10% and the rate of exposure (anti-HBc antibody positive) reaching up to 50% in blood donors and the general population [8-14].

HDV coinfects approximately 5% to 10% of individuals infected with HBV worldwide, although prevalence varies by geographic region [3,4]. Currently, epidemiological data for HDV are unavailable in Lao PDR.

The hepatitis B vaccine was first introduced in Lao PDR in 2001 at 6, 10, and 14 weeks of age (currently in the form of the pentavalent vaccine: diphtheria, tetanus, pertussis, hepatitis B, and *Haemophilus influenzae*). The birth dose within 24 hours was introduced to all newborns in Lao PDR in 2004 [13]. However, most adults, including health care workers (HCWs), were born before vaccine introduction.

HCWs are exposed to blood and body fluids due to their occupational activities and are therefore a high-risk group for HBV infection, with an infection rate up to 10 times higher than the general population [15]. Needle-stick injuries are the most frequently reported unsafe medical practices that lead to infection [16]. Other transmission routes are possible, such as from contaminated surfaces, as HBV also persists in the environment for up to 1 week [15,17-19]. The HBV transmission risk from HCWs to patients varies depending on the setting. Most published cases are in high-income countries, while evidence is often lacking for low-income countries [19,20]. HBV screening and vaccination before starting clinical practices are recommended to reduce the risk of disease transmission between HCWs and patients [21].

A previous study in 2013 found that Lao HCWs had low levels of seroprotection to HBV and low evidence of vaccination [9]. A similar serological profile was found for Lao dentists, who also had low knowledge and inadequate safety practices [11]. In 2019, the Lao Ministry of Health developed a strategic plan on combating the transmission of viral hepatitis by agreeing to provide health education and hepatitis B vaccination to susceptible groups including HCWs [22]. Despite this agreement and international recommendations [2], HBV vaccination for HCWs in Lao PDR is not routine yet. In this study, we aimed to estimate the exposure and seroprotection against HBV, as well as exposure to HDV, in Lao HCWs from 5 provinces, to provide guidance to key stakeholders such as the Lao Ministry of Health for HBV control decision-making.

## Methods

### Study Population

For this study, we used HCW serum samples collected in 2020 for the investigation of COVID-19 serology. The sample collection was detailed previously [23]. In brief, HCWs were recruited from central, provincial, and district hospitals in Vientiane (capital), Oudomxay, Luangprabang, Savannakhet, and Champasak provinces. Participants were randomly selected in each department and included clinicians, nurses, and laboratory technicians. We also included other nonclinician workers (administrative HCWs) in order to see if there was a trend in HBV exposure according to patient contact. After individual informed consent was obtained, a structured questionnaire was used to collect information on demographics and 5 mL of whole blood was collected. Serum was separated by centrifugation and stored at 4 °C for a maximum of 7 days and at −80 °C for long term.

### Ethical Considerations

Ethics approval was obtained from the Lao National Ethics Committee for Health Research (reference #052/2020). Informed consent was obtained from all participants involved in the study, and the consent forms from the study stated that any remaining serum might be used for testing serology against other infectious diseases. All participant data are deidentified, with personal identifiers removed and replaced by unique codes. Serum samples are stored securely, and access is restricted to authorized personnel only. Data are protected through encrypted storage and secure handling procedures. There was no compensation for the participants in the study according to the Lao National Ethic Committee regulations.

### Serology Testing

Anti-HBc and anti-HBs antibodies were measured by commercial enzyme-linked immunosorbent assays (ELISAs) according to the manufacturers’ instructions (Diasorin and Biorad). All anti-HBc–positive and anti-HBs–negative samples were tested for HBsAg (Biorad) [8]. All HBsAg-positive samples were tested for anti-HDV antibodies (indicating exposure to HDV) by ELISA (DIA.PRO). Individuals who were HBsAg positive were defined as having “acute or chronic infection,” individuals who were anti-HBc positive were defined as having been “previously exposed,” and individuals who were anti-HBc negative but anti-HBs positive were defined as having “serological evidence of vaccination.” Individuals who were anti-HBs negative had no serological evidence of protection and were defined as “susceptible to infection,” although it is possible that a small proportion were protected by the presence of memory B cells in the absence of detectable antibodies.

### Data Analysis

Descriptive statistics, bivariate analysis, and multivariable analysis were conducted using Stata (version 14; StataCorp). Descriptive statistics were used to summarize categorical and continuous variables. Bivariate analysis assessed the associations between the dependent variable (HBV serostatus) and independent variables (sociodemographics and work status). Results of the bivariate analyses are presented as crude odds ratios (cORs). Multivariable analysis, using logistic regression, was applied to variables with a *P* value ≤.20 from the bivariate analysis. Thus, in the final multivariate analysis model, the presence of anti-HBc antibody was compared with 4 factors: sex, age group, ethnicity, and province. No factors were found to be significantly linked to HBsAg positivity following bivariate analysis (all *P*>.20; Table 1). The serological profile of vaccination had only 2 variables in the multivariable analysis model: sex and age groups (Table 2). Both cORs and adjusted odds ratios (aORs) were reported from these analyses.

**Table 1.** Factors associated with anti-HBc^a^ and HBsAg^b^ positivity.

Factors	Anti-HBc positivity					HBsAg positivity		
	Participants, n/N (%)	Crude OR^c^ (95% CI)	*P *value	Adjusted OR (95% CI)	*P *value	Participants, n/N (%)	Crude OR (95% CI)	*P *value
**Total**	267/666 (40.1)	N/A^d^	N/A	N/A	N/A	36/666 (5.4)	N/A	N/A
**Sex**								
Male	65/148 (43.9)	1.0	N/A	N/A	N/A	12/148 (8.1)	1.0	N/A
Female	202/518 (39)	0.81 (0.56‐1.18)	.20	0.74 (0.50‐1.08)	.10	24/518 (4.6)	0.55 (0.26‐1.12)	.10
**Age group (years)**								
≤30	81/248 (32.7)	1.0	N/A	N/A	N/A	9/248 (3.6)	1.0	N/A
31‐49	120/300 (40)	1.37 (0.96‐1.95)	.07	1.42 (0.99‐2.04)	.054	19/300 (6.3)	1.79 (0.79‐4.04)	.10
≥50	66/118 (55.9)	2.61 (1.66‐4.10)	<.001^e^	2.91 (1.83‐4.61)	<.001^e^	8/118 (6.8)	1.93 (0.72‐5.13)	.10
**Ethnicity**								
Lao-Tai	254/643 (39.5)	1.0	N/A	N/A	N/A	36/643 (5.6)	1.0	N/A
Mone-Khmae	8/11 (72.7)	4.08 (1.07‐15.53)	.03^e^	2.31 (0.53‐10.10)	.20	0/11 (0)	—^f^	—
Hmong-Mien	5/12 (41.7)	1.09 (0.34‐3.48)	.80	0.69 (0.19‐2.54)	.50	0/12 (0)	—	—
**Province**								
Vientiane	160/410 (39)	1.0	N/A	N/A	N/A	26/410 (6.3)	1.0	N/A
Oudomxay	21/31 (67.7)	3.28 (1.50‐7.14)	.003^e^	3.69 (1.68‐8.12)	.001^e^	0/31 (0)	—	—
Luangprabang	19/47 (40.4)	1.06 (0.57‐1.96)	.80	1.05 (0.56‐1.98)	.80	1/47 (2.1)	0.32 (0.04‐2.42)	.20
Savannakhet	21/72 (29.2)	0.64 (0.37‐1.11)	.10	0.64 (0.36‐1.11)	.10	3/72 (4.2)	0.64 (0.18‐2.17)	.40
Champasak	46/106 (43.4)	1.19 (0.77‐1.84)	.40	1.13 (0.73‐1.77)	.50	6/106 (5.7)	0.88 (0.35‐2.21)	.70
**Year of service**								
≤10	123/357 (34.5)	1.0	N/A	N/A	N/A	17/357 (4.8)	1.0	N/A
11‐30	104/235 (44.3)	1.51 (1.07‐2.11)	.01	_	_	14/235 (5.9)	1.26 (0.61‐2.62)	.50
≥31	40/75 (54.1)	2.23 (1.34‐3.71)	.002^e^	_	_	5/74 (6.8)	1.44 (0.51‐4.06)	.40
**Contact status with patients**								
Indirect contact	23/46 (50)	1.0	N/A	N/A		4/46 (8.7)	1.0	N/A
Direct contact	244/620 (39.3)	0.64 (0.35‐1.18)	.10	_	_	32/620 (5.2)	0.57 (0.19‐1.69)	.30

a Anti-HBc: anti–hepatitis B core.

b HBsAg: hepatitis B surface antigen.

c OR: odds ratio.

d N/A: not applicable.

e *P*<.05.

f Variables that were not included in the multivariable analysis, that is, when *P*>.20 in the bivariate analysis.

**Table 2.** Factors associated with serological evidence of vaccination (anti-HBs^a^ positive and anti-HBc^b^ negative). Crude ORs^c^ are obtained from bivariate analysis and adjusted ORs are obtained from multivariable analysis.

Factors	Anti-HBs positive and anti-HBc negative				
	Participants, n/N (%)	Crude OR (95% CI)	*P* value	Adjusted OR (95% CI)	*P* value
**Total**	191/666 (28.7)	N/A^d^	N/A	N/A	N/A
**Sex**					
Male	37/148 (25)	1.0	N/A	N/A	N/A
Female	158/518 (29.7)	1.26 (0.83‐1.92)	.20	1.23 (0.80‐1.87)	.30
**Age group (years)**					
≤30	58/248 (23.4)	1.0	N/A	N/A	N/A
31‐49	104/300 (34.7)	1.73 (1.19‐2.53)	.004^e^	1.70 (1.16‐2.49)	.006^e^
≥50	29/118 (24.6)	1.06 (0.63‐1.78)	.80	1.03 (0.61‐1.73)	.08
**Ethnicity**					
Lao-Tai	186/643 (28.9)	1.0	N/A	N/A	N/A
Mone-Khmae	1/11 (9.1)	0.24 (0.03‐1.93)	.10	—^f^	—
Hmong-Mien	4/12 (33.3)	1.22 (0.36‐4.12)	.70	—	—
**Province**					
Vientiane	125/410 (30.5)	1.0	N/A	N/A	N/A
Oudomxay	5/31 (16.1)	0.43 (0.16‐1.16)	.09	—	—
Luangprabang	19/47 (40.4)	1.54 (0.83‐2.87)	.10	—	—
Savannakhet	19/72 (26.4)	0.81 (0.46‐1.43)	.40	—	—
Champasak	23/23 (21.7)	0.63 (0.38‐1.04)	.07	—	—
**Years of service**					
≤10	97/357 (27.2)	1.0	N/A	N/A	N/A
11‐30	72/235 (30.6)	1.18 (0.82‐1.70)	.30	—	—
≥31	22/74 (29.7)	1.13 (0.65‐1.96)	.60	—	—
**Contact status with patients**					
Indirect contact	12/46 (26.1)	1.0	N/A	N/A	N/A
Direct contact	179/620 (28.9)	1.15 (0.58‐2.27)	.60	—	—

a Anti-HBs: anti–hepatitis B surface.

b Anti-HBc: anti–hepatitis B core.

c OR: odds ratio.

d N/A: not applicable.

e *P*<.05.

f Variables that were not included in the multivariable analysis, that is, when *P*>.20 in the bivariate analysis.

## Results

### Population Characteristics

The total number of participants was 666. The median age was 34 (range 20-65) years, 77.8% (n=518) were female, and 96.5% (n=643) were of Lao-Tai ethnicity. Most participants were living in Vientiane capital (n=410, 61.6%) and 53.6% (n=357) had ≤10 years work experience. The majority of the participants had direct contact with patients (n=620, 93.1%; Table 3).

**Table 3.** Sociodemographic characteristics of participants.

Characteristics	Participants (N=666), n (%)	
**Total**	666 (100)	
**Sex**		
Male	148 (22.2)	
Female	518 (77.8)	
**Age group (years)**		
≤30	248 (37.2)	
31‐49	300 (45.1)	
≥50	118 (17.7)	
**Ethnicity**		
Lao-Tai	643 (96.5)	
Mone-Khmae	11 (1.7)	
Hmong-Mien	12 (1.8)	
**Province**		
Vientiane	410 (61.6)	
Oudomxay	31 (4.6)	
Luangprabang	47 (7.1)	
Savannakhet	72 (10.8)	
Champasak	106 (15.9)	
**Years of work**		
≤10	357 (53.6)	
11-30	235 (35.3)	
≥31	74 (11.1)	
**Contact status with patients**		
Indirect contact	46 (6.9)	
Direct contact	620 (93.1)	

### Hepatitis B and D Serology

Overall, 40.1% (267/666) HCWs were positive for anti-HBc antibodies (exposure), with no significant difference between male and female individuals (*P*=.10). HBV exposure in those aged ≤30 years was 32.7% (81/248), and the likelihood of the exposure was approximately 3 times higher in those aged ≥50 years (66/118, 55.9%; aOR 2.91, 95% CI 1.83‐4.61; *P*<.001). HCWs from Oudomxay province had higher HBV exposure than those living in Vientiane (21/31, 67.7% vs 160/410, 39%; aOR 3.69, 95% CI 1.68‐8.12; *P*=.001; Table 1 and MultimediaAppendices 1  2).

Overall, 36 (5.4% when extrapolated to the total study population) individuals were HBsAg positive (currently infected). The HBsAg prevalence was higher among men than women, although not significantly different (12/148, 8.1% and 24/518, 4.6%, respectively; cOR 0.48, 95% CI 0.26‐1.12; *P*=.10). HBsAg prevalence increased with age from 3.6% (9/248) in those aged ≤30 years to 6.8% (8/118) in those aged ≥50, although this also did not reach statistical significance (*P*=.10; Table 1 and Multimedia Appendix 1).

The overall prevalence of serological evidence for vaccination (anti-HBs positive and anti-HBc negative) was 28.7% (191/666). After multivariable analysis, individuals aged 31‐49 years had significantly higher odds (104/300, 34.7%; aOR 1.70, 95% CI 1.16‐2.49; *P*=.006) to have this profile than those aged ≤30 years (58/248, 23.4%; Table 2 and Multimedia Appendix 1).

A total of 284 (42.6%) participants were anti-HBs negative and susceptible to infection (Multimedia Appendix 3). All 36 HBsAg–positive samples tested negative for anti-HDV antibodies.

## Discussion

### Principal Findings

This study reported a moderate prevalence of 5.4% (36/666) HBsAg positivity among HCWs. This is similar to previous studies of Lao HCWs, with prevalence ranging from 3% to 12% [9,11]. However, it is lower than the prevalence in the adult general population (8%‐10%) [8,13], perhaps due to the fact that two-thirds of the participants were female individuals, who have lower exposure and HBsAg prevalence as compared to male individuals [11,14,24,25]. Indeed, in this study, male HCWs had higher prevalence of HBsAg positivity than female HCWs, although this was not statistically significant. This difference could be due to differential immune response, hormones, or risks practices [26,27]. Also, we found that HBsAg positivity increased with age. This is similar to what we reported previously in HCWs and the general population [9,24]. Our data show that a substantial proportion of Lao HCWs are chronically infected with HBV, and therefore, appropriate precautions are needed to reduce the risk of onward transmission to patients. All HCWs should be tested for HBsAg, and HCWs who are HBsAg positive should get access to counseling and treatment. Furthermore, the high proportion of HCWs with no serological evidence of protection indicates that the health care facilities need to implement routine HBV vaccination, with 3 doses and boosters as necessary for all HCWs. After receiving the hepatitis B vaccine, anti-HBs immunoglobulin G levels can remain detectable for several years, usually lasting a minimum of 5 to 10 years, and in some cases, even longer [5].

None of the individuals who were HBsAg positive had anti-HDV antibodies in this study, suggesting that HDV prevalence might be low in Lao PDR. However, since HDV prevalence varies with geographical regions and study cohorts (eg, in neighboring Vietnam, the prevalence ranged from 0% to 43% among individuals who are HBsAg positive [28-31], and in Thailand, from 0% to 65% [32-34]), further studies are needed to determine whether there are any subpopulations with HDV exposure in Lao PDR.

Anti-HBc seroprevalence, indicating exposure, was 40.1% (267/666). This high level is similar to other studies in Lao PDR [8,10,13]. As expected, HBV exposure increased with age. This was in accordance with previous published studies in the Lao general population [8,12,13,25,35] and HCWs [9,11] and may reflect exposure in the workplace or elsewhere. There was no correlation of exposure with the length of time in the job or type of job, as reported in a previous study of Lao HCWs [9]. However, the distinction between staff with and without contact with patients was not always clear, as some administration staff may have previously worked in a clinical setting. HBV exposure was higher in those living in the northern provinces. In particular, HCWs from Oudomxay province had significantly higher exposure than HCWs from other provinces. The data agreed with studies of HBV in Lao blood donors, where significantly higher exposure was found in the northern provinces [14,35]. The reason for this geographical variation is not clear. The rate of HBsAg positivity was not significantly higher in the northern provinces in this study. This may be due to the lower proportion of participants from the northern provinces in this study and the overall lower numbers of individuals who were HBsAg positive; due to HBV infection happening during adulthood, when chronic infection is less likely to occur; or a combination of both reasons.

This study found a low percentage of HCWs who had the serological evidence of vaccination (191/666, 28.7%). This profile was more common in those 31‐49 years of age, suggesting that this age group has been vaccinated against HBV as an adult or perhaps during an ad hoc HBV vaccination campaign in their hospitals. Although the rate of participants with serological evidence of vaccination in this study is higher than other studies conducted among HCWs in Lao PDR (13.6% [11] and 21% [9]) and Indonesia (11.6%) [36], it remains inadequate and reveals a large number of HCWs without protective anti-HBs antibodies (31.2%; Multimedia Appendix 3). Importantly, many of the participants with anti-HBc and anti-HBs antibodies may also have been vaccinated, but there is no way to distinguish these from the individuals exposed to HBV without vaccination based on the laboratory results.

This study has several limitations. First, we did not comprehensively measure the risk factors and practices of the participants. Understanding the intensity of exposure and the nature of contact with patients could provide valuable insights into the factors contributing to HBV exposure among HCWs. Second, the reason for higher HBV exposure in the northern populations has not been addressed—an issue that could facilitate effective management strategies. Lastly, the sample size and locations were limited. A more comprehensive, nationwide survey is needed at all health facilities in order to establish HBV testing, treatment, and vaccination practices as well as serological profiles.

### Conclusions

According to the findings of this study, HBsAg prevalence is intermediate with no evidence of HDV coinfection in HCWs in Lao PDR. Importantly, a high proportion of HCWs remains susceptible to infection. Therefore, there is a need to implement mandatory pre-employment hepatitis B testing, in accordance with the *Lao National Strategic Plan on Viral Hepatitis 2024‐2030*. This would reduce the risk of HBV infection for both HCWs and patients. The obstacles to implementation of routine, comprehensive nationwide testing, vaccination, and treatment for HCWs include low awareness and lack of financial and human resources. These issues require an investment in HCW training, financial investment, and political will. Furthermore, the high proportion of HCWs with no serological evidence of protection indicates that the health care facilities need to implement routine HBV vaccination, with 3 doses and boosters as necessary for all HCWs.

## Supplementary material

10.2196/65093 Multimedia Appendix 1 Hepatitis B serology by age group. **P≤.01, ***P≤.001.

10.2196/65093 Multimedia Appendix 2 Hepatitis B serology by province. ***P≤.001.

10.2196/65093 Multimedia Appendix 3 Serological profile of participants.

## Abbreviations

Anti-HBc: anti–hepatitis B core
Anti-HBs: anti–hepatitis B surface
aOR: adjusted odds ratio
cOR: crude odds ratio
ELISA: enzyme-linked immunosorbent assay
HBsAg: hepatitis B surface antigen
HBV: hepatitis B virus
HCW: health care worker
HDV: hepatitis D virus
Lao PDR: Lao People’s Democratic Republic
WHO: World Health Organization
