# Supplementary figures and images for: Hepatitis B Virus Exposure, Seroprotection Status, and Susceptibility in Health Care Workers From Lao People’s Democratic Republic: Cross-Sectional Study
Source: JMIR Public Health Surveill. 2024 Dec 17;10:e65093. doi: 10.2196/65093 (PMC11683653; doi:10.2196/65093)

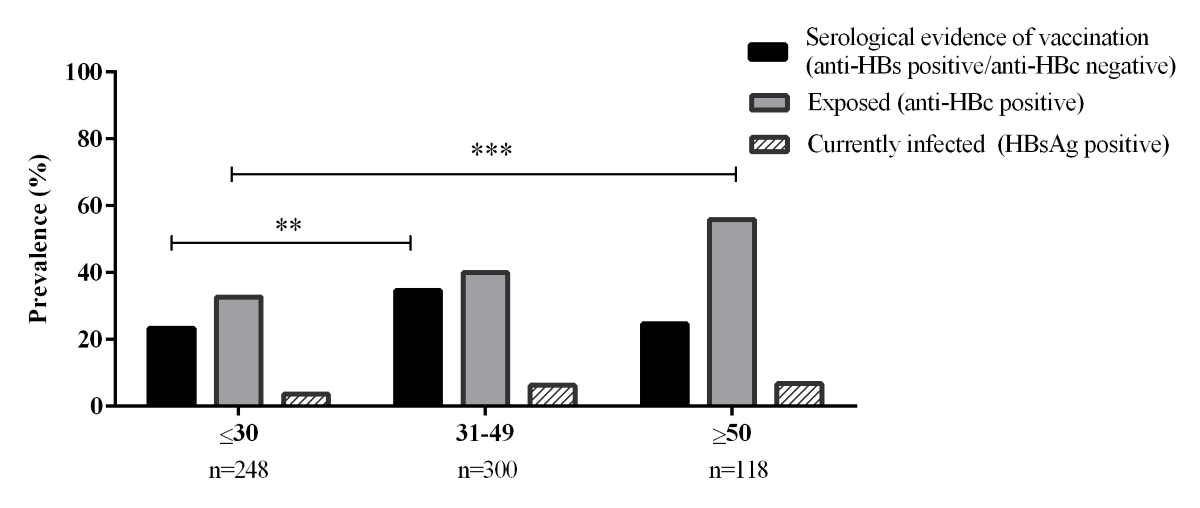

Supplement: Multimedia Appendix 1 [file publichealth-v10-e65093-s001.png]

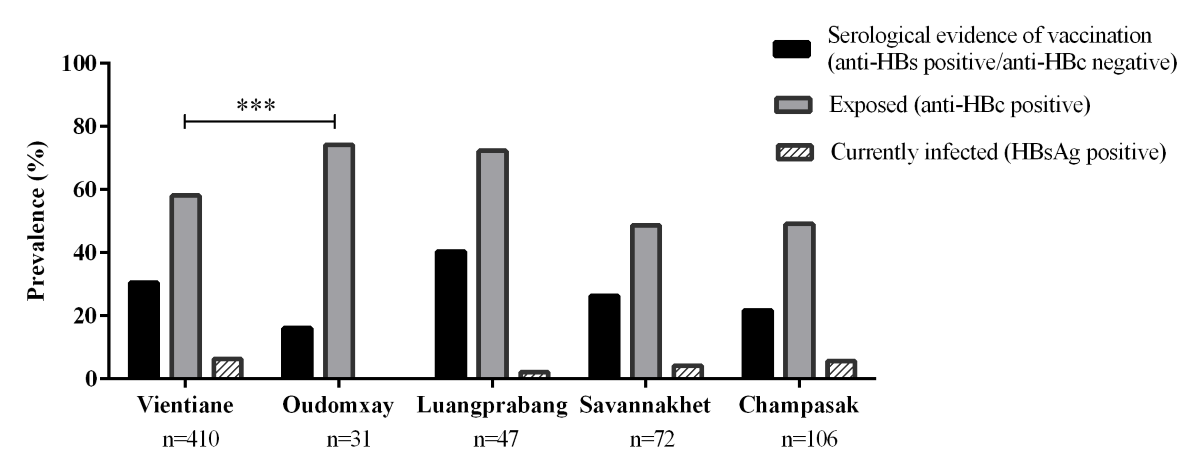

Supplement: Multimedia Appendix 2 [file publichealth-v10-e65093-s002.png]
