# Supplementary material for: Hepatitis B Virus Exposure, Seroprotection Status, and Susceptibility in Health Care Workers From Lao People’s Democratic Republic: Cross-Sectional Study
Source: JMIR Public Health Surveill. 2024 Dec 17;10:e65093. doi: 10.2196/65093 (PMC11683653; doi:10.2196/65093)
Supplement: Multimedia Appendix 3 [file publichealth-v10-e65093-s003.docx]

**Supplementary table**

Table S1 Serological profile of participants

| **Serological profile** | **n** | **%** | **Interpretation** |
| --- | --- | --- | --- |
| anti-HBs negative/anti-HBc negative | 208 | 31.2 | No HBV infection– susceptible |
| anti-HBs positive/anti-HBc negative | 191 | 28.7 | Serological evidence of vaccination |
| anti-HBs positive/anti-HBc positive | 191 | 28.7 | Previous HBV infection |
| anti-HBs negative/anti-HBc positive/HBsAg negative | 40 | 6.0 | Previous HBV infection |
| anti-HBs negative/anti-HBc positive/HBsAg positive | 36 | 5.4 | Current HBV infection |
